# *Bacillus*-Mediated Silver Nanoparticle Synthesis and Its Antagonistic Activity against Bacterial and Fungal Pathogens

**DOI:** 10.3390/antibiotics10111334

**Published:** 2021-11-01

**Authors:** Nivedhitha Kabeerdass, Ahmed Al Otaibi, Manikandan Rajendran, Ayyar Manikandan, Heba A. Kashmery, Mohammed M. Rahman, P. Madhu, Anish Khan, Abdullah M. Asiri, Maghimaa Mathanmohun

**Affiliations:** 1 Department of Microbiology, Muthayammal College of Arts & Science, Rasipuram, Namakkal DT 637408, Tamil Nadu, India; maheshnive30@gmail.com; 2 Chemistry Department, Faculty of Science, University of Ha’il, P.O. Box 2440, Ha’il 81451, Saudi Arabia; ahmed.alotaibi@uoh.edu.sa; 3 Department of Biotechnology, Padmavani Arts and Science College for Women, Salem 636011, Tamil Nadu, India; rvmani.85@gmail.com; 4 Department of Chemistry, Bharath Institute of Higher Education and Research (BIHER), Bharath University, Chennai 600073, Tamil Nadu, India; manikandana.che@bharathuniv.ac.in; 5 Centre for Catalysis and Renewable Energy, Bharath Institute of Higher Education and Research (BIHER), Bharath University, Chennai 600073, Tamil Nadu, India; 6 Chemistry Department, Faculty of Science, King Abdulaziz University, Jeddah 21589, Saudi Arabia; hkashmery@kau.edu.sa (H.A.K.); mmrahman@kau.edu.sa (M.M.R.); asiri2@gmail.com (A.M.A.); 7 Center of Excellence for Advanced Materials Research, King Abdulaziz University, Jeddah 21589, Saudi Arabia; 8 Department of Mechanical Engineering, Malnad College of Engineering, Hassan, Visvesvaraya Technological University, Belagavi 590018, Karnataka, India; madhu.p.gowda15@gmail.com

**Keywords:** antibacterial, antifungal, AgNPs, paddy plant growth promotion

## Abstract

In this article, the supernatant of the soil-borne pathogen *Bacillus* mn14 was used as the catalyst for the synthesis of AgNPs. The antibacterial and antifungal activity of Bs-AgNPs was evaluated, in which *S. viridans* and *R. solani* showed susceptibility at 70 µL and 100 µL concentrations. Enzyme properties of the isolates, according to minimal inhibitory action and a growth-enhancing hormone–indole acetic acid (IAA) study of the isolates, were expressed in TLC as a purple color with an Rf value of 0.7. UV/Vis spectroscopy revealed the presence of small-sized AgNPs, with a surface plasmon resonance (SPR) peak at 450 nm. The particle size analyzer identified the average diameter of the particles as 40.2 nm. The X-ray diffraction study confirmed the crystalline nature and face-centered cubic type of the silver nanoparticle. Scanning electron microscopy characterized the globular, small, round shape of the silver nanoparticle. AFM revealed the two-dimensional topology of the silver nanoparticle with a characteristic size ranging around 50 nm. Confocal microscopy showed the cell-wall disruption of *S. viridans* treated with Bs-AgNPs. High-content screening and compound microscopy revealed the destruction of mycelia of *R. solani* after exposure to Bs-AgNPs. Furthermore, the Bs-AgNPs cured sheath blight disease by reducing lesion length and enhancing root and shoot length in *Oryza sativa* seeds. This soil-borne pathogen *Bacillus*-mediated synthesis approach of AgNPs appears to be cost-efficient, ecofriendly, and farmer-friendly, representing an easy way of providing valuable nutritious edibles in the future.

## 1. Introduction

The distinctive physicochemical properties of AgNPs have attracted the attention of the technical community [1], e.g., their elevated thermal conductivity, chemical permanence, and antibacterial effects [2]. Nanotechnology and nanoscale materials have emerged as potent delivery methods for several ailments. The fabulous potential applications of microbial-mediated synthesized nanoparticles toward microbial multidrug resistance and microbial biofilm creation [3] are considered an alternative to germicidal agents [4]. The antimicrobial activities of microbial-mediated synthesized AgNPs are impressive [5]. The synthesis of microbial-mediated synthesized AgNPs aligns well with green chemistry values, and the viridescent fusion of AgNPs results in an environmentally friendly material which is nonhazardous to all living organisms [6]. The metal microparticles are synthesized by bioremediation, which is an ecofriendly substitute for fermentation techniques. The natural and artificial (chemical) products facilitate living status. Biogenically synthesized AgNPs less than 100 nm in size have distinctive physical, chemical, and biological characteristics and a broad variety of applications in the fields of pharmaceuticals and medicine, such as diagnosis and treatment of cardiovascular diseases, wound healing, implantable biomaterials, drug delivery, molecular imaging, purification, fabrics, inhibitory response to inflammation, viral infections, angiogenesis, and platelet activities. Bacterial cell walls impregnated with thick and thin layers of polysaccharides and fungi were observed to enhance chemotherapeutic activities by grouping with biogenic AgNPs. The environment and climatic cycle are also beneficial for good yielding. Good yielding depends on the plantation field featuring biosynthetic organics compressing metal nanoparticles [7,8]. *Bacillus* species combined with silver nanoparticles enhance nutrient uptake capacity; accordingly, this microorganism possesses valid economic importance due to its inhibitory biotic and abiotic effects in plantation fields, characterized as a plant growth-promoting rhizobacterium. The routine use of agricultural products has led to the development of hybrids. Metal nanoparticles play a major role in marketing hybridized agricultural products [9]. Beneficial microbes in mineralized plantation fields can synthesize metal nanoparticles, which enhance the healthy botanical floral geotropism of the phenetic and pharmacological sectors. Hence, this research work aimed to execute the green synthesis of silver nanoparticles from *Bacillus* sp. Of the rhizosphere and to investigate their antifungal and antibacterial action toward human pathogens.

## 2. Methodological Analysis

### 2.1. Isolation of Rhizosphere Bacillus

Twenty-seven roots of *Oryza sativa* plants procured from the rice fields in and around Rasipuram were aseptically uprooted, followed by surface sterilization with 10% sodium hypochlorite solution and Milli-Q water. After collection, the soil was transferred to the laboratory in sterile polythene bags for further processing. Then, the soil was finely sieved to separate unwanted materials and weighed. The commensals in the soil were identified by serial dilution using 0.8% NaCl, followed by plating in nutrient agar to identify particular isolates [10].

### 2.2. Phenotypic Identification

After determining the size, shape, color, and texture of the colony from the nutrient agar plate after incubation, the contents were lightly rubbed on a slide and partially exposed to a Bunsen burner flame for heat fixing. Then, the slide coated with isolates underwent Gram staining. A further confirmatory study of the morphology of the isolates was conducted using Kovacs reagent, methyl red, glucose broth, and hydrogen peroxide in appropriate quantities, added dropwise to the nutrient broth containing the isolates [11]. A positive result of this biochemical test gives us an idea about the sample’s physiological status, which facilitates further processing.

### 2.3. Growth-Promoting Assay and Enzyme Activity

#### 2.3.1. Growth-Promoting Characteristics of the Isolate (IAA Siderophore and Phosphate Solubilization)

##### IAA

Bacterial isolates were inoculated at 37 °C for 24 h in freshly prepared nutrient broth containing 0.3% tryptophan and then vortexed at 120 rpm. Next, Salkowski’s reagent was added dropwise, before incubating for 30 min; the color change after incubation was determined on the basis of the OD value. Then, to identify the compound, a TLC slide was prepared using silica gel with acetic acid and benzene in a 4:1 ratio. Then, the sample and IAA were spotted on the TLC plate, and the color was recorded after spraying the spot with ninhydrin reagent, before calculating the Rf value [12].

##### Siderophore

To detect the siderophore production, bacterial isolates were inoculated in Chrome Azurol S (CAS) medium containing malt extract and incubated at 30 °C for 72 h. After incubation, an orange or light-orange halo was obtained, indicating the production of siderophores [13].

##### Phosphate Solubilization

The pure bacterial culture was inoculated in freshly prepared Pikovskaya’s agar medium, and the plates were incubated for 48 h. The clearance zone was observed and measured after incubation.

#### 2.3.2. Enzyme-Synthesizing Characteristics of the Isolates (Protease, Lipase, Amylase, and Pectinase)

The bacterial colonies were inoculated in freshly prepared pectin, carboxymethyl cellulose, protease, and starch plates and incubated at 37 °C for 24 h. After incubation, formation of a halo indicated the enzyme-producing properties of the isolates [14].

### 2.4. Synthesis of Silver Nanoparticles

The selected isolate was inoculated in 1000 mL of sterile freshly prepared nutrient broth [11]. Then, the flask was placed in a rotating shaker (REMI, RS.24BL 11/07, India) at 200 rpm and incubated for 48 h at room temperature. Next, the culture was centrifuged at 12,000 rpm for 10 min [12], before separating the supernatant and mixing it with a sterile AgNO_3_ solution at various concentrations (1 mM, 2 mM, and 5 mM) [15]. These mixtures were returned to the rotating shaker at 200 rpm and then incubated for periods of 6 h, 12 h, and 36 h, along with the control. They were periodically checked for a color change indicating the formation of silver nanoparticles before further characterization [14,15,16]. 

### 2.5. Collection of Bacterial Samples

Gram-positive and Gram-negative pathogens such as *Salmonella typhi*, *S. viridans*, *Staphylococcus aureus*, *S. epidermis*, *Candida albicans*, *Klebsiella pneumonia*, *Pseudomonas aeruginosa*, *Bacillus cereus*, and *Escherichia coli* were aseptically inoculated in sealed containers. These pathogens were procured from the laboratory in Rasipuram, transported to the college laboratory, and stored in a refrigerator [16,17]. Later, they were subcultured in appropriate media, e.g., blood agar, nutrient agar, Eosin methylene agar, and MacConkey agar. After incubation and morphological observation, the plates were neatly sealed, labeled, and stored at −2 °C before the evaluation of antimicrobial activity [18].

### 2.6. Collection of Fungal Samples

*Rhizoctonia solani*, *Macrophomina phaseolina*, and *Sclerotium rolfsii* were procured in an aseptically sealed PDA plate from the laboratory in Rasipuram. Then, all pathogens were subcultured in appropriate media and maintained at room temperature for further evaluation of antagonistic activity [19].

### 2.7. Antagonistic Activity

#### 2.7.1. Antibacterial Study

The bacterial culture of each organism was applied evenly to freshly prepared Mueller–Hinton agar plates using a sterile swab. Then, a plug was made on each plate using a sterile cork boret (6 mm). Synthesized Bs-AgNPs were loaded in various concentrations (5 µL, 10 µL, 20 µL, 30 µL, 40 µL, 50 µL, 60 µL, and 70 µL) in each well using a sterile micropipette tip [20,21]. All plates were labeled and placed in an upright position in the incubator (Ausco incubator, India).

#### 2.7.2. Antifungal Study

After 48 h, the fungal cultures were equally spread on Potato Dextrose Agar using a sterile swab tip. Then, after 2–3 days of incubation, a plug was made on each plate using a sterile cork boret (6 mm). Different concentrations of synthesized Bs-AgNPs were loaded using a 100 µL pipette. Then, the plates were labeled and maintained aseptically in an upright position at 37 °C (room temperature) [22].

### 2.8. Analytical Characterization

The formation of metallic ions was confirmed by naked eye through a color transformation from light brown to dark brown; the optical characteristics were studied using a UV/Vis spectrophotometer (Systronics sys119, India). Frequently wiped dust-free quartz tubes were used to house samples and blank solutions (water or solvents such as acetone). The instrument software enabled visualizing the sample/blank name, molar concentration, frequency, time, and temperature on a monitor. The same process was repeated for all samples at diverse time intervals [23].

A particle size analyzer was used to determine the size range of the particles in the powdered sample using laser diffractometry (DeXel-Filtrate TM Optical, India) [24]. This instrument measures the particle refractive index and particle absorption coefficient over 70 cycles in 10 s. The size and distribution of the silver dispersions in the quartz tube were examined.

The crystalline structure of the synthesized AgNPs was investigated using XRD (D8 advance Eco XRD systems with SSD160 1 D detector Bruker). The diffraction pattern of the particle was noted. Samples used were first pulverized before adding small volumes of polypropylene and polyethylene to the grinding jar, followed by small volumes of halogens 30 min later. Then, the average particle size was determined using a graph [25].

Atomic force microscopy (AFM) (Nanosurf AFM, Switzerland) is an influential tool for the determination of particle topography, and it works according to Hooke’s law. A small volume of the sample is taken and spotted on a neat coverslip, which is mounted on the AFM stub and dried at 37 °C. A minimum of five images are obtained using a silicon probe cantilever [26].

SEM (TSCAN (Floor Model, US) was used to obtain a three-dimensional image of the synthesized silver nanoparticles [27]. The samples were pretreated with solvents to remove oil before fixing with phosphates or glutaraldehyde. They were dehydrated using alcohol or acetone, followed by complete drying in a vacuum environment. Finally, the specimens were mounted and observed under the microscope, and images were captured by the camera.

### 2.9. Instrumental Analysis for the Bacterial and Fungal Study

#### 2.9.1. Confocal Microscopy for Bacterial Activity

The bacterial samples showing positive results in the antibacterial assay were treated with Bs-AgNPs and incubated for 2 h. Then, the samples were sealed under coverslips. Later, a drop of acridine orange dye was added before incubating in the dark for 10 min [28]. The sample was mounted on a slide in a paper reinforcement ring, sealed using a coverslip, and varnished using nail polish.

#### 2.9.2. High-Content Screening Study for Fungal Activity

High-content screening produces quantitative data of the cells. The potent and effective fungal samples were co-inoculated with *Bacillus* species mn14-mediated AgNPs (Bs-AgNPs) in potato dextrose broth and kept in a rotatory shaker for 3 days. After 48 h, the mycelia were fixed in 4% formalin. Then, 100 µL of mycelium was added dropwise onto a clean slide, and a drop of lactophenol cotton blue stain was added before transferring to a 96-well flat-bottom plate [29]. The samples were treated with 5 µL/mL Hoechst 33258, and the cells were incubated for 15 min in the dark, followed by processing in a high content screening system.

#### 2.9.3. Compound Microscopy Study for Fungal Activity

The fungal and *Bacillus*-mediated AgNP interactions were studied by pressing in the samples onto a coverslip and then adding a drop of lactophenol cotton blue for visualization under a compound microscope [30,31].

## 3. Results

### 3.1. Phenotypic Study

Phenotypically, among the 27 potential bacterial isolates, *Bacillus* species mn14 was the most potent. Its structure, size, and shape were determined, and it was identified as a Gram-positive organism. The positive result of the biochemical test confirmed it as a *Bacillus* species.

#### 3.1.1. Growth-Promoting and Enzymatic Characteristics of the Isolate

**Figure 1:**
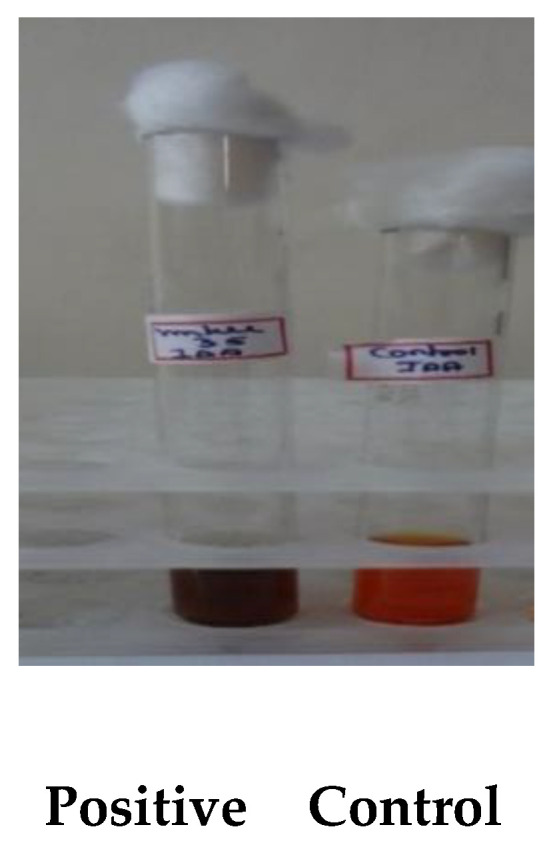
Indole acetic acid (IAA).

**Figure 2:**
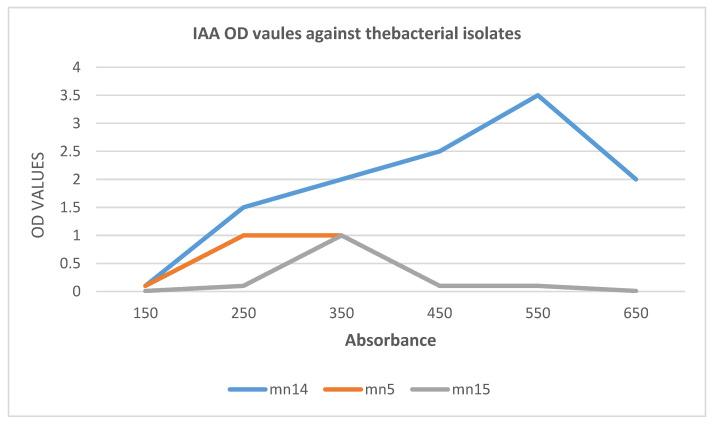
IAA OD values vs. bacterial isolates.

Among the 27 isolates *Bacillus* mn14, mn5, and mn15 exhibited positive results for IAA, whereas no color change was observed for siderophore and phosphate solubilization. The OD values were recorded and a peak was observed at a wavelength of 578 nm; the compound was identified by subjecting the mixture to TLC in a ratio of 4:1 acetic acid and benzene. A light-purple spot was later identified by spraying with ninhydrin, and the Rf value (distance traveled by the solvent) was determined. The results are presented in Table 1 and Figure 1 and Figure 2.

Figure 1 shows that BS-mn14 has the ability to produce IAA is indicated by the colour change from orange to dark brown.

Among the 27 isolates, *Bacillus* mn14 showed effective activity against IAA, as well as moderate activity in the case of siderophore and phosphate solubilization.

Figure 2 shows that the absorbance wavelength of *Bacillus* mn14 was 550 nm, in contrast to mn5 and mn15 with wavelengths in the range of 240–350 nm.

#### 3.1.2. Enzymatic Characteristics of the Isolates

*Bacillus* mn14 showed inhibition zones of 17 mm for protease, 16 mm for amylase, 16 mm for pectinase, and 19 mm for cellulase. The other isolates showed less or no activity toward these enzymes, as shown in Table 2.

The enzymatic properties of the 27 *Bacillus* isolates were examined using protease, amylase, pectinase, and cellulase assays. Among these *Bacillus* species, the mn14 isolate alone revealed inhibitory concentrations in all cases.

### 3.2. Biosynthesis of Silver Nanoparticles

**Figure 3:**
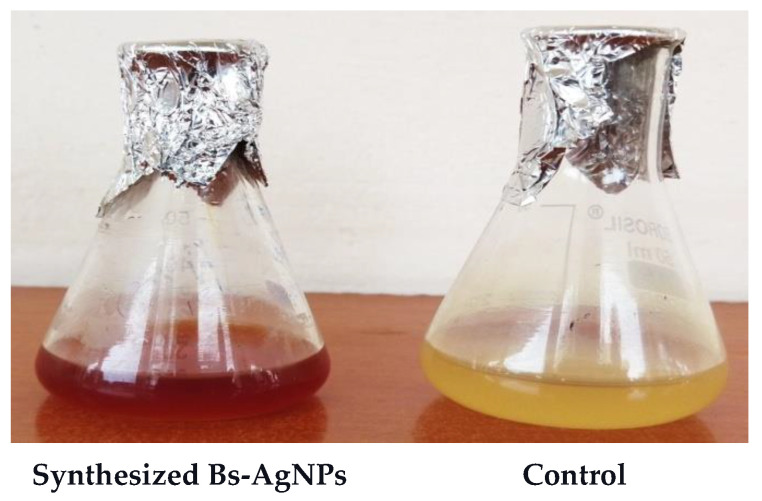
*Bacillus* mn14-mediated silver nanoparticles (Bs-AgNPs).

Pale-yellow to chocolate-brown AgNPs were obtained at 5 mM concentration after 24 h of incubation, biosynthesized from the biomass of *Bacillus* species mn14 in the absence of light, thus yielding Bs-AgNPs, as shown in Figure 3.

### 3.3. Antibacterial Activity

The synthesized *Bacillus mn14*-AgNPs (Bs-AgNPs) and the positive control tetracycline were introduced to Gram-positive and Gram-negative organisms. Using Bs-AgNPs, *S. viridans* exhibited an inhibition zone of 20 mm at 70 µL, *S. aureus* exhibited an inhibition zone of 18 mm at 70 µL, and *C. albicans* exhibited an inhibition zone of 18 mm at 70 µL, which were larger than the zones obtained in the case of tetracycline at 70 µL (positive control), as shown in Table 3.

### 3.4. Antifungal Activity

**Figure 4:**
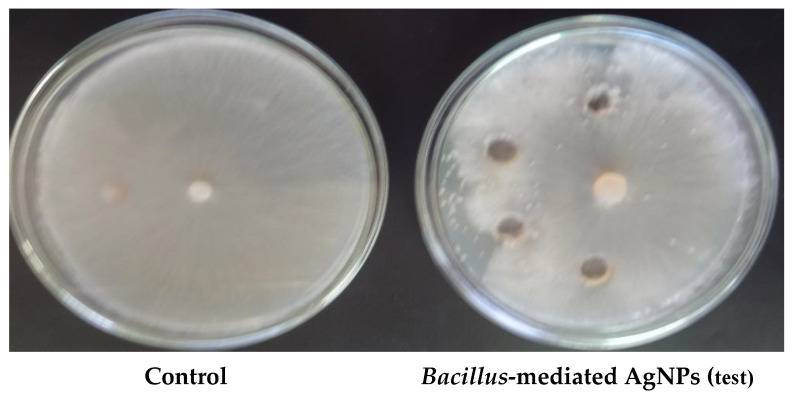
Antifungal activity of *Bacillus* species-mediated AgNPs (Bs-AgNPs) against *R. solani*.

Three fungal pathogens, *R. solani*, *M. phaseolina*, and *S. rolfsii*, were exposed to the bacterial isolates to identify antagonistic activity. *R. solani* showed a maximum inhibition zone of 23 mm in the presence of *Bacillus* mn14, compared with other isolates. The other two fungal pathogens showed less or no activity. *R. solani* treated with BS-AgNPs exhibited an inhibition zone of 20 mm at 100 µL (lower compared with tetracycline), suggesting that *Bacillus mn14*-AgNPs might act as a nano fungicide. The inhibition zones of other isolates are shown in Table 4 and Table 5 and Figure 4.

Three soil-borne fungal pathogens, *R. solani*, *M. phaseolina*, and *S. rolfsii*, were tested against 27 *Bacillus* species isolated from the rhizosphere soil of an *Oryza sativa* field. Among these isolates, mn14 showed elevated susceptibility to *R. solani* compared with the other two pathogens, *M. phaseolina* and *S. rolfsii*, which exhibited moderate susceptibility and resistant activity.

The *R. solani* soil-borne fungal pathogen which showed effective antagonistic activity was inoculated with different concentrations of Bs-AgNPs and carbendazim (control). Bs-AgNPs showed a minimum inhibition zone of 20 mm at 100 µL, compared to carbendazim with an inhibition zone of 15 mm at 100 µL.

### 3.5. UV Absorption Spectra

**Figure 5:**
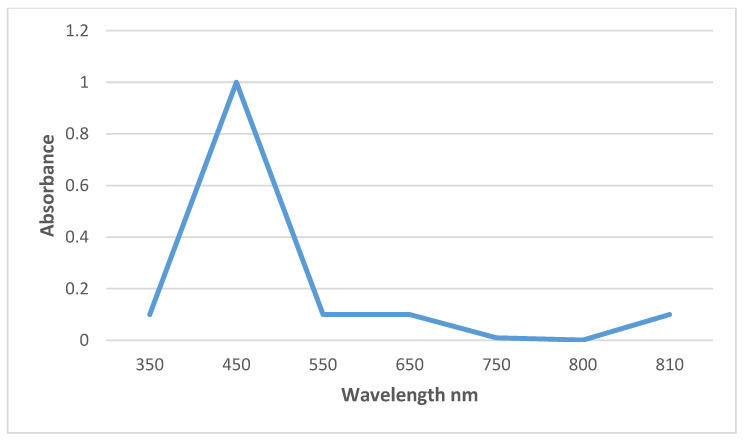
UV/Vis spectrum. The surface plasmon resonance peak of the Bs-AgNP solution observed at 450 nm confirmed the synthesis of small, polydisperse silver nanoparticles.

In the synthesized Bs-AgNP solution, the reduction of AgNO_3_ to silver nanoparticles and its stability were ascertained through UV absorption spectra. After incubation, a slight narrow surface plasmon resonance peak was observed at 350 nm, indicating the formation of small-sized silver nanoparticles. Bands at longer wavelengths with peaks of surface plasmon resonance observed at 450 nm after incubation indicated the formation of unevenly shaped, polydisperse silver nanoparticles; the absorbance corresponding to wavelength is shown in Figure 5.

### 3.6. Particle Size Analyzer

**Figure 6:**
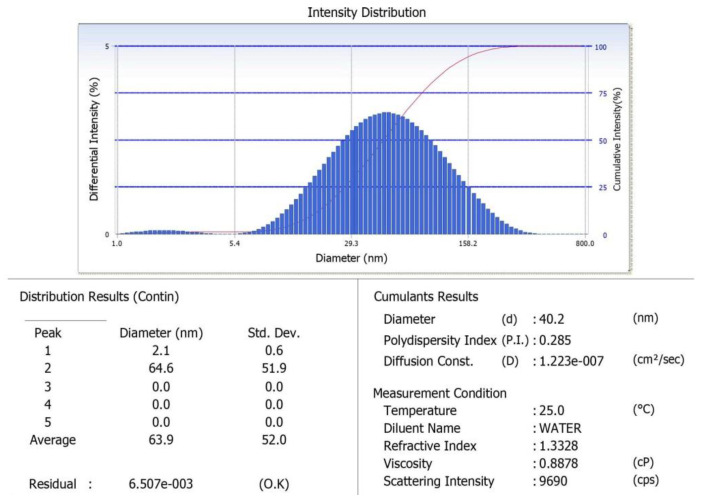
Particle size analysis. The synthesized *Bacillus subtilis* mediated AgNPs showed an average diameter of 40.2 nm with a refractive index of 0.285 and a viscosity of 0.8878 at 25 °C.

The average diameter of the synthesized AgNPs was 40.2 nm, with a refractive index of 1.3 and viscosity of 0.88 at 25 °C; the scattering intensity, polydispersity index, and diffusion constant values are also schematically presented in Figure 6.

### 3.7. XRD

**Figure 7:**
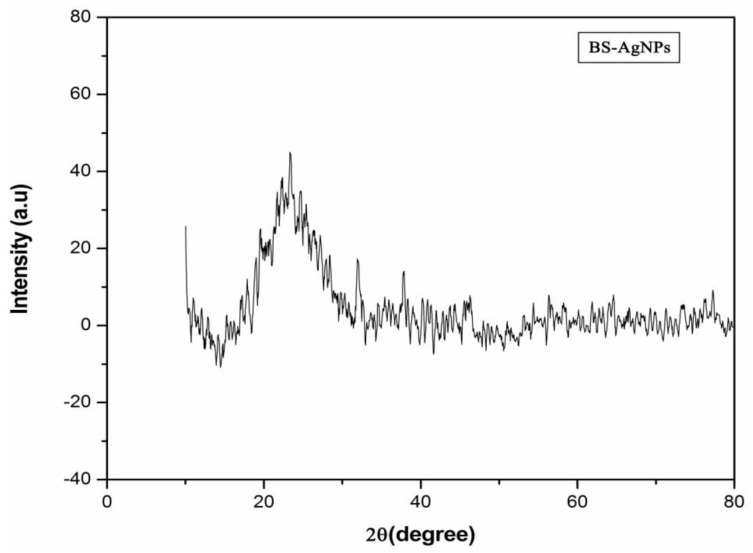
XRD. X-ray diffraction analysis showing the characteristic crystalline nature of the silver nanoparticles, illustrating the diffraction peaks corresponding to their intensity.

The diffraction peaks of (111), (200), (220), and (311) at 2θ = 38.27°,46.27°, 64.64°, and 77.66° due to Bragg reflection reveal the cubic lattice crystal-like formation of silver nanoparticles. The existence of silver and the presence of crystallized bio-organic compounds on the surface of the AgNPs are shown in Figure 7.

### 3.8. Scanning Electron Microscopy

**Figure 8:**
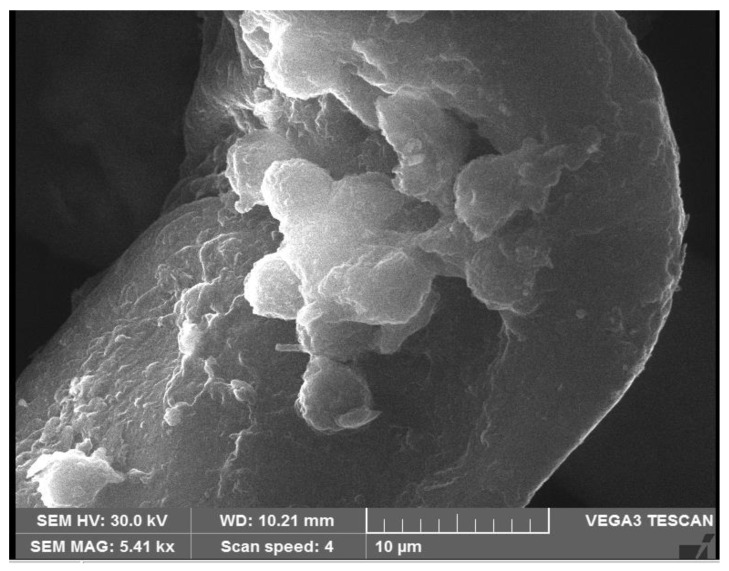
Scanning electron micrograph showing the synthesized AgNPs coated to the organic phase, viewed at 10 µm resolution, with a size of 40 nm at a magnification of 5400×.

Scanning electron microscopy allowed capturing an image of the particle at a resolution of 10 µm. The *Bacillus* mn14-mediated silver nanoparticles were round, spherical, globular, curved, and small ball-shaped. The size of the Bs-AgNPs was determined to be 40 nm size at a magnification of 5400×, as shown in Figure 8.

#### 3.8.1. Atomic Force Microscopy

**Figure 9:**
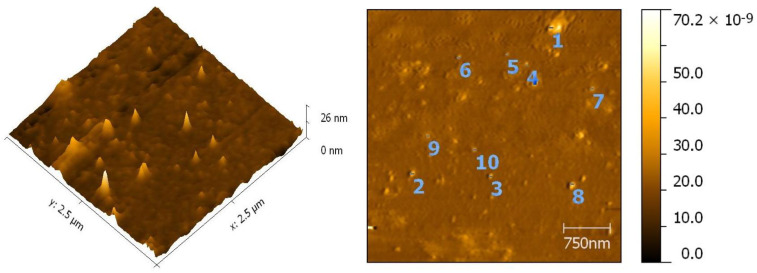
Atomic force micrograph showing the two-dimensional image of the synthesized AgNPs with an average size of 50 nm.

Atomic force microscopy confirmed the shape and size of the silver nanoparticles. Symmetrical, spherical-shaped nanoparticles were dispersed uniformly with a particle size of approximately 50 nm, as shown in Figure 9.

#### 3.8.2. Instrumental Analysis for Bacterial and Fungal Study

##### Confocal Microscopy for Bacterial Activity

**Figure 10:**
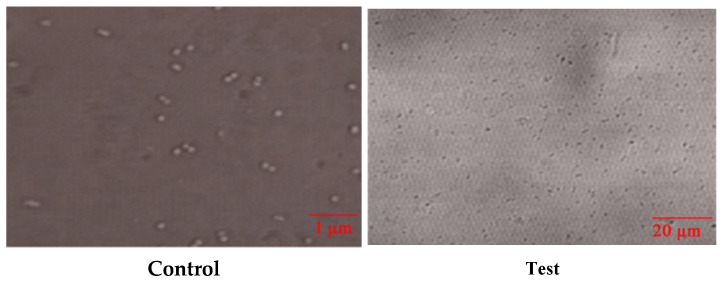
Confocal micrographs with control showing the presence of live cells, whereas the cells treated with *Bacillus*-mediated silver nanoparticles were destroyed.

The bacterial cells treated with Bs-AgNPs were observed under a microscope to differentiate live and dead cells as a function of dye absorption. The control slide received no treatment, showing only the presence of the bacterial cells. Destruction of the cell wall can be observed in the test slide shown in Figure 10.

**Figure 11:**
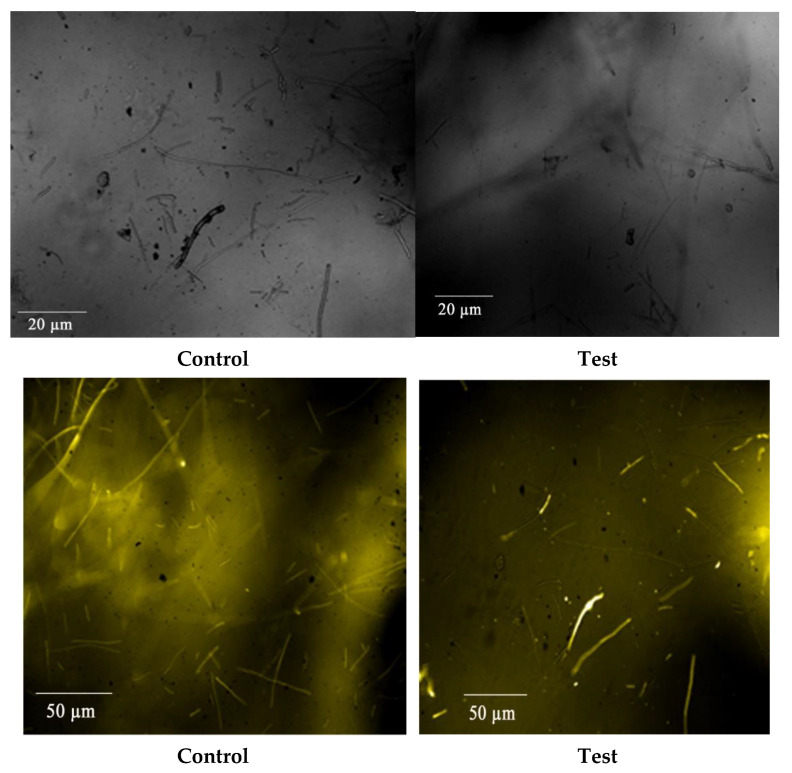
High-content screening images showing the presence of the mycelia in the control slide and treatment slide, where Bs-AgNPs resulted in the destruction of the cell wall and mycelia of *R. solani*.

Among the three fungal pathogens, the *R. solani* showed a greater inhibition zone at 100 µL concentration than carbendazim. The cell wall of *R. solani* treated with Bs-AgNPs was viewed under a microscope, showing its disruption according to high-content screening (Figure 11).

#### 3.8.3. Compound Microscopy for Fungal Activity

**Figure 12:**
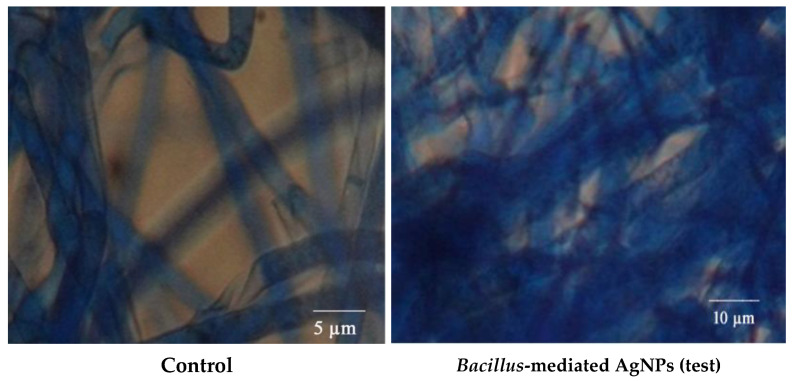
Fungal activity study by a compound microscope.

After treating *R. solani* with Bs-AgNP solution, the mycelia were viewed under an ordinary compound microscope. Destruction of the mycelia can be seen in Figure 12 when compared with carbendazim (control).

#### 3.8.4. Applications of Synthesized Bs-AgNPs in Treating Leaves Affected by Sheath Blight Disease

**Figure 13:**
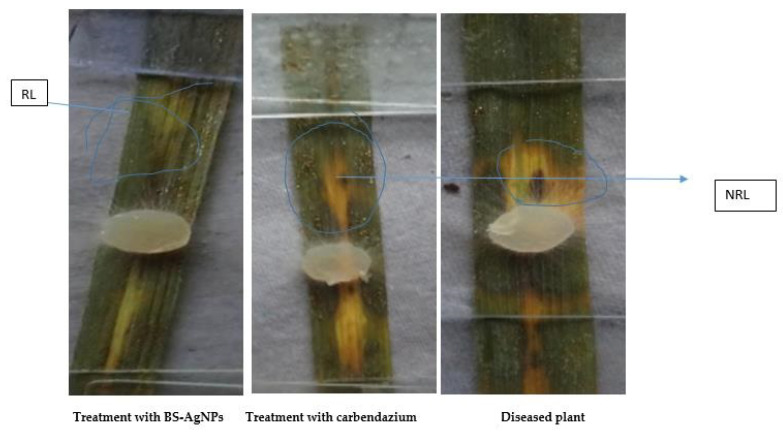
Leaves affected by sheath blight disease treated with Bs-AgNPs or carbendazim, exhibiting a reduction or increase in lesion length, respectively, highlighting the potential application of *Bacillus*-mediated silver nanoparticles as a nano fungicide. RL, reduced lesion; NRL, no reduction in lesion.

Diseased leaves were treated with Bs-AgNP solution and kept for observation. After a few weeks, there was a reduction in lesion length when compared with the fungicide carbendazim (control), as shown in Table 6 and Figure 13.

Three *Oryza sativa* leaf samples affected by sheath blight disease were taken and treated with Bs-AgNPs or fungicide carbendazim (control). After 14 days, a reduction in lesion length from 8–4.6 cm was noted in leaf sample 1 treated with Bs-AgNPs, whereas no reduction was seen when exposed to carbendazim (8 cm). The lesion length was reduced to 3.2 cm in leaf sample 2 when exposed to Bs-AgNPs compared to the 6 cm obtained in the presence of carbendazim. The lesion length was completely reduced in leaf sample 3 when treated with Bs-AgNPs.

#### 3.8.5. Growth Promotion of *Oryza sativa* Seeds

**Figure 14:**
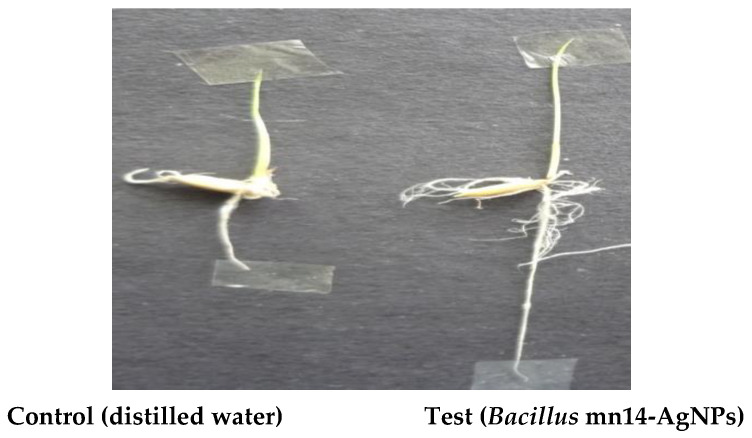
Plant growth promotion.

Seeds of *Oryza sativa* were collected from the local market. Seeds were kept in dry conditions in the dark. Surface sterilization was conducting by immersion in 10% sodium hypochlorite solution for a few minutes. The control seed was treated with water. The germination process was initiated by placing a piece of tissue paper in a Petri dish, before adding 5 mL of *Bacillus* mn14-mediated AgNPs and incubating. In each experiment, the control seeds were taken for comparison with the treated ones, and the germination process on a daily basis is detailed in Table 7 and Figure 14.

A few *Oryza sativa* seeds were treated with distilled water (control) or Bs-AgNPs, with the former showing a root length of 1.5 cm and shoot length of 1.54 cm and the latter showing an elevated root length of 5.42 cm and an elevated shoot length of 2.08 cm. The vigor index was also elevated in the case of Bs-AgNPs, confirming their positive effect on seed germination.

## 4. Discussion

In this study, bio nanoparticles synthesized in the microbial supernatant of *Bacillus* mn14 enabled the generation of AgNPs as confirmed by a color change. A reduction of AgNPs to silver after exposure of the supernatant enriched with bacterial cells to a minimal concentration of AgNO_3_ (5 mM) exhibited a color change from straw to chocolate brown within 12 h. The above-stated protocol for AgNP synthesis is time-saving, ecofriendly, and cost-effective, being completed within 12 h using a soil-borne pathogen. The antagonistic activity of *Bacillus* mn14 toward human pathogens was investigated, with *S. viridans* showing a zone of 20 mm at 70 µL concentration, suggesting that Bs mn14 might act as a bactericidal agent. Fungal pathogen the *R. solani* showed a minimum inhibition zone of 19 mm at 100 µL concentration, suggesting that Bs mn14 might also act as an antifungal or fungicidal agent. Enzyme activity and growth-enhancing assays exposed the beneficial characteristics of this isolate toward the production of crops [32,33]. These properties push it to the forefront of nanotechnology research. UV/Vis spectrophotometry provided further confirmation with an SPR peak at 450 nm [34,35,36]. The average diameter of the spherical-shaped silver nanoparticles was 40.2 according to particle size analysis. The X-ray diffraction analysis peaks at 38.27°, 46.27°, 64.64°, and 77.66° corresponding to planes (111), (200), (220), and (311) revealed the cubic lattice crystal-like formation of silver nanoparticles. Scanning electron microscopy allowed identifying the small balloon-shaped, globular structure of silver nanoparticles at a magnification of 5400×. The texture of the synthesized nanoparticles and their agglomeration were easily visualized using AFM, revealing a characteristic size of approximately 50 nm. Confocal microscopy allowed observing disruption of the bacterial cell wall, confirming the potential application of this silver nanoparticle as a nano bactericidal agent. In the case of fungi, pretreatment with *Bacillus*-mediated silver nanoparticles showed a 70% destruction of the mycelia according to high-content screening and compound microscopy, suggesting an additional role as a therapeutic agent in curing diseased foliage affected by sheath blight disease [37,38,39,40]. Post-treatment showed a reduction in lesion length when compared with the fungicide. Enhanced *Oryza sativa* seed germination and elevated vigor index after treatment with Bs-AgNPs suggest their potential as a nano fertilizer. Attachment of Ag^+^ ions to the negatively charged cell surface results in inhibiting the generation of ATP from ADP, disrupting the ionic and aqueous balance within a cell, hindering the passage of molecules across the biological barrier, and rendering the molecule susceptible to reactive oxygen species. The exposure of proteins, DNA, sulfur, and other cell constituents to *Bacillus*-mediated silver nanoparticles can lead to apoptosis.

## 5. Conclusions

This reduction of silver nitrate to silver ions via the addition of an oxygen atom covalently bonded to the hydrogen and oxygen atoms of extracellular peptides leads to the production of silver nanoparticles [41,42,43,44,45,46]. Stable monodisperse AgNPs of different shapes are able to coat proteins in the enriched organic phase. The synthesized nanoparticles showed excellent antagonistic activity toward the human pathogen *S. viridans*, along with beneficial effects in treating lesions in diseased leaf and in increasing the shoot and root length of a rice plant, highlighting the multiple roles of the synthesized silver nanoparticles as an antibacterial [47], antifungal, and growth-promoting agent. As an ecofriendly and cheap option in the nanotechnology field, *Bacillus*-mediated silver nanoparticles may be considered nano fertilizers, nano fungicides, and nano bactericidal agents.

## Figures and Tables

**Table 1:** Enzymatic characterization of the *Bacillus* isolates.

S.No.	Isolates	IAA	Siderophore	Phosphate Solubilization
1.	mn1–3	Negative	Negative	Negative
2.	mn6–12	Negative	Negative	Negative
3.	mn13–27	Positive	Positive	Positive

**Table 2:** Enzymatic characteristics of the isolates.

S.No.	Isolates	Zone of Inhibition			
		Protease	Amylase	Pectinase	Cellulase
1.	mn1	11 mm	7 mm	Nil	Nil
2.	mn3	10 mm	10 mm	Nil	5 mm
3.	mn4	10 mm	Nil	5 mm	Nil
4.	mn5, 6, 8	10 mm	Nil	Nil	Nil
5.	mn9, 23	10 mm	6 mm	Nil	Nil
6.	mn10	Nil	9 mm	Nil	Nil
7.	mn11	13 mm	Nil	Nil	Nil
8.	mn12	5 mm	10 mm	Nil	Nil
9.	mn15	7 mm	5 mm	Nil	Nil
10	mn16	8 mm	11 mm	Nil	Nil
11.	mn14	17 mm	16 mm	16 mm	19 mm
12.	mn20	3 mm	Nil	Nil	Nil
13.	mn21	4 mm	Nil	Nil	Nil
14.	mn2, 7, 13, 19, 17, 18, 22, 24, 25, 26, 27	Nil	Nil	Nil	Nil

**Table 3:** Antibacterial activity of synthesized Bs-AgNPs vs. tetracycline (control) against human pathogens.

S. No.	Human Pathogen	Inhibition Zome (mm) of Bs-AgNPs against Human Pathogens								Inhibition Zone (mm) of Tetracycline (Control) against Human Pathogens							
		5 µL	10 µL	20 µL	30 µL	40 µL	50 µL	60 µL	70 µL	5 µL	10 µL	20 µL	30 µL	40 µL	50 µL	60 µL	70 µL
1.	*S. typhi*	5 ± 0.25	5 ± 0.25	5 ± 0.25	5 ± 0.25	6 ± 0.25	6 ± 0.25	6 ± 0.25	6 ± 0.25	6 ± 0.25	6 ± 0.25	6 ± 0.25	6 ± 0.25	6 ± 0.25	6 ± 0.25	6 ± 0.25	6 ± 0.25
2.	*S. viridans*	15 ± 0.25	16 ± 0.25	16 ± 0.25	16 ± 0.25	16.9 ± 0.25	20 ± 0.25	17.5 ± 0.25	20 ± 0.25	15 ± 0.25	16 ± 0.25	16 ± 0.25	16 ± 0.25	16.9 ± 0.25	16 ± 0.25	17.5 ± 0.25	18 ± 0.25
3.	*S. aureus*	10 ± 0.25	11 ± 0.25	12 ± 0.25	15 ± 0.25	15 ± 0.25	15 ± 0.25	15 ± 0.25	18 ± 0.25	10 ± 0.25	11 ± 0.25	12 ± 0.25	13 ± 0.25	16 ± 0.25	17 ± 0.25	15 ± 0.25	17 ± 0.25
4.	*S. epidermis*	6.8 ± 0.25	6.8 ± 0.25	8 ± 0.25	7..2 ± 0.25	7.2 ± 0.25	7.8. ± 0.25	8.3 ± 0.25	8.3 ± 0.25	6.8 ± 0.25	6.8 ± 0.25	6.8 ± 0.25	7 ± 0.25	7.2 ± 0.25	7.2 ± 0.25	8.3 ± 0.25	8.3 ± 0.25
5.	*C. albicans*	10 ± 0.25	12 ± 0.25	14 ± 0.25	15 ± 0.25	16 ± 0.25	18 ± 0.25	19 ± 0.25	18 ± 0.25	10 ± 0.25	12 ± 0.25	14 ± 0.25	15 ± 0.25	16 ± 0.25	16 ± 0.25	19 ± 0.25	18 ± 0.25
6.	*K. pneumoniae*	6 ± 0.25	6 ± 0.25	6 ± 0.25	6 ± 0.25	7 ± 0.25	8 ± 0.25	9 ± 0.25	9 ± 0.25	6 ± 0.25	6 ± 0.25	6 ± 0.25	6 ± 0.25	6 ± 0.25	6 ± 0.25	9 ± 0.25	9 ± 0.25
7.	*P. aeruginosa*	9 ± 0.25	12 ± 0.25	12 ± 0.25	14 ± 0.25	15 ± 0.25	15 ± 0.25	16 ± 0.25	15 ± 0.25	9 ± 0.25	12 ± 0.25	12 ± 0.25	14 ± 0.25	15 ± 0.25	15 ± 0.25	16 ± 0.25	14 ± 0.25
8.	*B.cereus*	12 ± 0.25	13 ± 0.25	13 ± 0.25	13 ± 0.25	14 ± 0.25	14 ± 0.25	16 ± 0.25	15 ± 0.25	12 ± 0.25	12 ± 0.25	12 ± 0.25	13 ± 0.25	14 ± 0.25	14 ± 0.25	16 ± 0.25	15 ± 0.25
9.	*E. coli*	10 ± 0.25	12 ± 0.25	13 ± 0.25	14 ± 0.25	14 ± 0.25	14 ± 0.25	15 ± 0.25	15 ± 0.25	10 ± 0.25	12 ± 0.25	13 ± 0.25	13 ± 0.25	14 ± 0.25	14 ± 0.25	15 ± 0.25	14 ± 0.25

Antagonistic activity of human pathogens (Gram-positive and Gram-negative) after treatment with Bs-AgNPs at various concentrations or antibiotic tetracycline (control). *S. viridans* showed elevated susceptibility (20 mm) at 70 µL concentration to BS-AgNPs compared with tetracycline. The above data also show that Gram-positive organisms were more susceptible than Gram-negative organisms.

**Table 4:** Antagonistic activity of *Bacillus* species toward three fungal pathogens.

S.No.	Isolates of *Bacillus* sp.	Zone of Inhibition in mm		
		*R. solani*	*M. phaseolina*	*S. rolfsii*
1.	mn1	6.5 ± 0.25	9 ± 0.25	7 ± 0.25
2.	mn2	7.2 ± 0.25	Nil	Nil
3.	mn3	4.5 ± 0.25	Nil	Nil
4.	mn4	4.5 ± 0.25	Nil	Nil
5.	mn5, 8	6.0 ± 0.25	Nil	Nil
6.	mn6	6.0 ± 0.25	7 ± 0.25	Nil
7.	mn7, 27	7.0 ± 0.25	Nil	Nil
8.	mn9	11.0 ± 0.25	Nil	Nil
9.	mn10	15.0 ± 0.25	9 ± 0.25	Nil
10.	mn11	12.0 ± 0.25	Nil	5 ± 0.25
11.	mn12	20.0 ± 0.25	6 ± 0.25	9 ± 0.25
12.	mn13	19.0 ± 0.25	8 ± 0.25	Nil
13.	mn14	23.0 ± 0.25	9 ± 0.25	6 ± 0.25
14.	mn15	17.0 ± 0.25	5 ± 0.25	7 ± 0.25
15.	mn16	19.0 ± 0.25	Nil	11 ± 0.25
16.	mn17	11.0 ± 0.25	Nil	Nil
17.	mn19	8.0 ± 0.25	Nil	Nil
18.	mn20	11.0 ± 0.25	Nil	Nil
19.	mn18, 21, 22, 23, 24, 25	9.0 ± 0.25	Nil	Nil
20.	mn24, 26	8.0 ± 0.25	Nil	Nil

**Table 5:** Characteristic features of the isolates.

S.No.	Concentration	Inhibition Zone in mm										
		5 µL	10 µL	20 µL	30 µL	40 µL	50 µL	60 µL	70 µL	80 µL	90 µL	100 µL
1.	*Bacillus*-mediated AgNPs (Bs-AgNPs)	6 ± 0.25	7 ± 0.25	8 ± 0.25	9 ± 0.25	9 ± 0.25	10.5 ± 0.25	15 ± 0.25	15 ± 0.25	14 ± 0.25	15.5 ± 0.25	20 ± 0.25
2.	Carbendazim	5 ± 0.25	4 ± 0.25	4 ± 0.25	5 ± 0.25	5 ± 0.25	7 ± 0.25	13 ± 0.25	13 ± 0.25	14 ± 0.25	14 ± 0.25	15± 0.25

**Table 6:** Lesion length of the diseased leaf.

Diseased Leaf Sample	Treatment with Bs-AgNPs (Lesion Length)	Treatment with Fungicide (Carbendazim) (Lesion Length)
Diseased sample leaf 1	4.6 cm	8 cm
Diseased Sample leaf 2	3.2 cm	6 cm
Diseased sample leaf3	Nil	3.1 cm

**Table 7:** *Oryza sativa* seed germination after treatment with Bs-AgNPs.

S.No.	Treatment	Germination (%)	Root Length(cm)	Shoot Length (cm)	Dry Weight (g)	Vigor Index
1.	Distilled water (control)	100	1.5	1.54	0.0203	1552.8
2.	Bs-AgNPs (*Bacillus* mediated AgNPs	90	5.42	2.08	0.0399	2415.6

## Data Availability

Not applicable.
